# Increased seroreactivity to HERV-K10 peptides in patients with HTLV myelopathy

**DOI:** 10.1186/1743-422X-10-360

**Published:** 2013-12-23

**Authors:** Raisa Perzova, Elliot Graziano, Swathi Sanghi, Caitlin Welch, Patricia Benz, Lynn Abbott, Danielle Lalone, Jordan Glaser, Thomas Loughran, William Sheremata, Bernard J Poiesz

**Affiliations:** 1 Department of Medicine, Division of Hematology/Oncology, State University of New York, Upstate Medical University, Syracuse, NY 13210, USA; 2 Division of Infectious Diseases, Department of Medicine, Staten Island Hospital, New York, NY, USA; 3 Penn State Cancer Institute, Penn State Milton S. Hershey Medical Center, Hershey, PA, USA; 4 Department of Neurology, University of Miami, Miami, FL, USA

## Abstract

**Background:**

Previously, we had shown that persons infected with human T-cell lymphoma leukemia virus 1 or 2 (HTLV-1 or 2) had an increased prevalence of antibodies to a peptide in the Pol protein of the retrovirus HERV-K10, homologous to a peptide in HTLV gp21 envelope protein. The prevalence rate was higher in those with myelopathy vs. non-myelopathy. We have now extended our observations to a cohort restricted to North America in whom the diagnosis of HTLV myelopathy was rigorously confirmed to also test for reactivity to another HERV-K10 peptide homologous to the HTLV p24 Gag protein.

**Methods:**

Sera from 100 volunteer blood donors (VBD), 53 patients with large granular lymphocytic leukemia (LGLL), 74 subjects with HTLV-1 or 2 infection (58 non-myelopathy and 16 myelopathy) and 83 patients with multiple sclerosis (MS) were evaluated in ELISA assays using the above peptides.

**Results:**

The HTLV myelopathy patients had a statistically significant increased prevalence of antibodies to both HERV-K10 peptides (87.5%) vs. the VBD (0%), LGLL patients (0%), MS patients (4.8%), and the HTLV positive non-myelopathy subjects (5.2%).

**Conclusion:**

The data suggest that immuno-cross-reactivity to HERV-K10 peptides and/or transactivation of HERV-K10 expression by the HTLV Tax protein may be involved in the pathogenesis of HTLV-associated myelopathy/tropical spastic paraparesis and spastic ataxia.

## Introduction

In addition to causing T-cell malignancies, both human T-cell lymphoma/leukemia viruses 1 and 2 (HTLV-1 and HTLV-2) are known to cause myelopathy (HAM) in a minority of infected individuals [1-6]. The complete pathogeneses of these chronic, clinically variable, but sometimes severe, life threatening neurologic disorders are not completely understood. However, compared to asymptomatic HTLV infected individuals, patients with HAM have a higher HTLV proviral DNA load, higher rates of HTLV virus production and higher titers of anti-HTLV antibodies and cytotoxic T-cells [7]. Early in the disease, areas of central nervous system involvement show high numbers of polyclonal HTLV infected T-lymphocytes and an intense polyclonal uninfected cellular immunologic response [7-12]. HTLV-1 tends to primarily affect the thoracic spinal cord and is characterized by progressive spastic paraparesis, while HTLV-2 is skewed toward the cerebellum and is characterized by ataxia and usually a milder clinical course [6].

**Figure 1:**
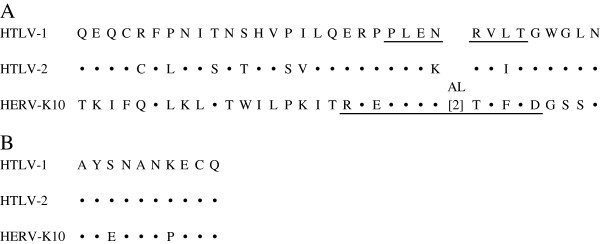
**Alignment of amino acid sequences homologous to HTLV-1 BA21 Env peptide (A) and HTLV-1 p24 Gag peptide (B).** Corresponding areas of conservation in HTLV-2 Env and HERV-K10 Pol or HTLV-2 p24 Gag and HERV-K10 Gag are indicated by the (•) symbol. Amino acid changes are as shown. The underlined peptide in HTLV-1 Env had been previously mapped to be the target of anti-HTLV-1 p21 Env seroreactivity in LGLL patients. The underlined peptide in HERV-K10 Pol and that shown for HERV-K10 Gag were used in the studies described herein. The GenBank accession numbers for various sequences are: HTLV-1 Env AAA96674; HTLV-2 Env AAZ16506; HERV-K10 Pol P10266; HTLV-1 Gag AAA85325; HTLV-2 Gag P14077, and HERV-K10 P87889.

In previous studies we had determined that, although only 8% of patients with large granular lymphocytic leukemia (LGLL) were infected with HTLV-2, almost half the patients had antibodies to HTLV p24 Gag and gp21 Env proteins [13]. None of these patients were infected with HTLV-1, HTLV-3, HTLV-4, or bovine leukemia virus. The seroreactivity to the HTLV gp21 Env protein was mapped to an eight amino acid peptide (Figure 1). The only other life form or human amino acid sequence in the available data bases with homology to both HTLV p24 Gag and the relevant eight amino acid sequence in HTLV gp21 Env was the endogenous human retrovirus HERV-K10 (Figure 1). Accordingly, we tested volunteer blood donors (VBD), LGLL patients and HTLV-1 or HTLV-2 infected individuals, some of whom had myelopathy, for antibodies to a peptide in HERV-K10 Pol that was homologous to the HTLV-1 gp21 Env peptide. While there was a slight, statistically significant increased prevalence of anti-HERV-K10 Pol peptide antibodies in the LGLL group vs VBD, the more interesting observations were that the HTLV infected population had a statistically significant higher prevalence of antibodies to HERV-K10 Pol peptide than the VBD, and that this prevalence was significantly skewed to those patients with myelopathy. Herein, to obviate against any cross-reactivity secondary to polyconal gammopathies that are known to be prevalent in more underdeveloped parts of the world, we investigated these observations further in a cohort of subjects from the continental United States and Canada. Also, we restricted the study to those in whom the diagnosis of HTLV myelopathy was rigorously confirmed. In addition to testing for antibodies to the aforementioned, HERV-K10 Pol peptide, we also tested for antibodies to a HERV-K10 Gag peptide which is homologous to HTLV p24 Gag protein (Figure 1).

We also did molecular studies involving HERV-K113 and HERV-K115, which are members of the HERV-K10 family [14]. They have been integrated into the human genome multiple times after humans separated into races and ethnic groups. Because they are present in polymorphisms among humans, are capable of producing almost a complete repertoire of viral proteins, and have a higher prevalence in certain human autoimmune diseases, we analyzed for their presence among the study populations.

## Results

**Figure 2:**
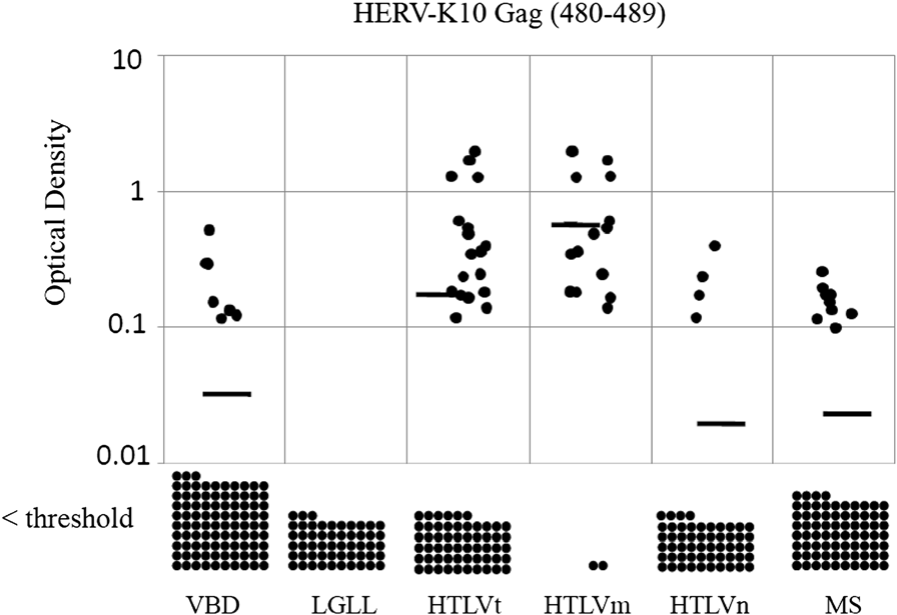
**Distribution of optical density minus background readings for the anti-HERV-K10 Gag peptide ELISA antibody assay performed at a dilution of 1:200 among the various subject populations.** The horizontal bars represent the means for each group and each dot represents one subject. Values below the threshold for positive are shown at the bottom. The means for VBD, LGLL, HTLVt, HTLVm, HTLVn and MS subjects were 0.027, 0, 0.142, 0.598, 0.016 and 0.017, respectively. VBD vs. LGLL, p = 0.002; VBD vs. HTLVt, p = < 0.001; VBD vs. HTLVm, p = < 0.001; VBD vs. HTLVn, p = < 0.001; VBD vs. MS, p = 0.331; HTLVm vs. HTLVn, p = < 0.001.

**Figure 3:**
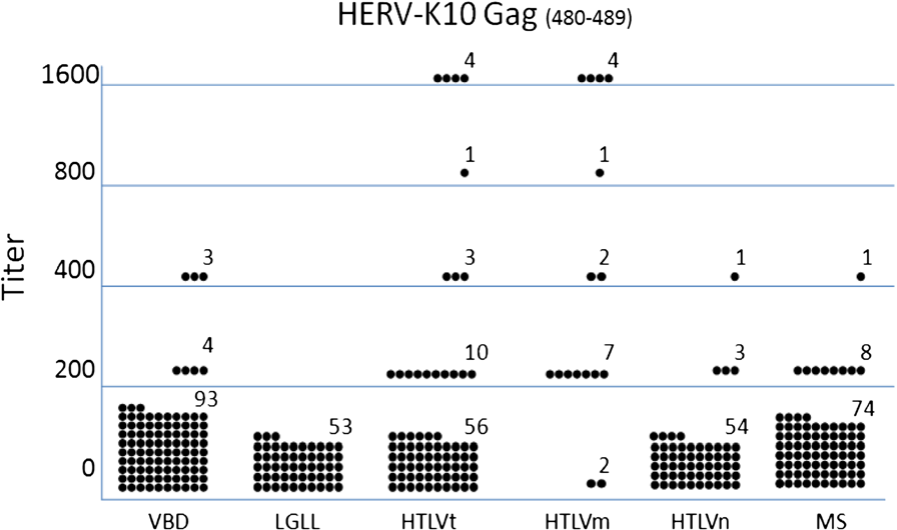
**Distribution of seropositive titers for the anti-HERV-K10 Gag peptide ELISA antibody assay among the various subject populations.** The means for VBD, LGLL, HTLVt, HTLVm, HTLVn and MS subjects were 94, 0, 154, 613, 28, 101, respectively. VBD vs. LGLL, p = 0.002; VBD vs. HTLVt, p = 0.002; VBD vs. HTLVm, p = < 0.001; VBD vs. HTLVn, p = < 0.001; VBD vs. MS, p = 0.550; HTLVm vs. HTLVn, p = < 0.001.

Table 1 shows the prevalence rates for anti-HERV-K10 Gag and Pol antibodies among the various subject populations. The LGLL patients (0%) had significantly lower anti-HERV-K10 Gag seroprevalence rates than the VBD (7%), suggesting that this peptide may not be the target of the previously mentioned anti-HTLV p24 Gag reactivity observed in LGLL patients. The anti-Gag reactivity of the VBD was not significantly different than that observed in the HTLVn (7%) and MS subjects (11%). However, the HTLVm patients had a significantly higher seroprevalence rate (88%) than all the other groups. These same relationships were maintained when one compares the distributions of optical density or antibody titer for anti-HERV-K10 Gag antibodies among the various populations (Figure 2, Figure 3).

**Table 1:** Prevalence of antibodies to HERV-K10 Gag and Pol peptides in different populations

	**VBD**		**LGLL**		**HTLVt**		**HTLVm**		**HTLVn**		**MS**	
Peptides	#tested	#pos (%)	#tested	#pos (%)	#tested	#pos (%)	#tested	#pos (%)	#tested	#pos (%)	#tested	#pos (%)
HERV-K10 Gag*	100	7(7)	53	0(0)	74	18(24)	16	14(88)	58	4(7)	83	9(11)
HERV-K10 Pol^+^	100	0(0)	53	3(6)	74	21(28)	16	15(94)	58	6(10)	83	13(16)

*HERV-K10 Gag VBD vs. LGLL, p = 0.047; VBD vs. all HTLV+, p = 0.001; VBD vs. HTLV + myelopathy, p = < 0.001: VBD vs. HTLV + non-myelopathy, p = 0.626; VBD vs. multiple sclerosis, p = 0.256; HTLV + myelopathy vs. HTLV + non-myelopathy, p = <0.001; HTLV + myelopathy vs. MS p = < 0.001.

^+^HERV-K10 Pol VBD vs. LGLL, p = 0.040; VBD vs. all HTLV+, p = 0.001; VBD vs. HTLV + myelopathy, p = < 0.001: VBD vs. HTLV + non-myelopathy, p = 0.002; VBD vs. multiple sclerosis, p = < 0.001; HTLV + myelopathy vs. HTLV + non-myelopathy, p = <0.001; HTLV + myelopathy vs. MS, p = < 0.001.

**Figure 4:**
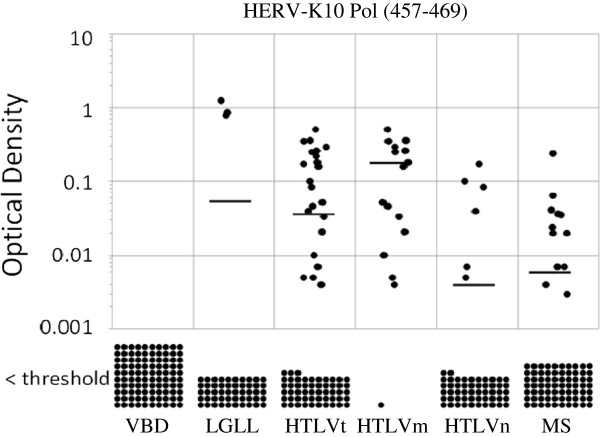
**Distribution of optical density minus background readings for the anti-HERV-K10 Pol peptide ELISA antibody assay performed at a dilution of 1:200 among the various populations.** The means for VBD, LGLL, HTLVt, HTLVm, HTLVn and MS subjects were 0, 0.054, 0.037, 0.128, 0.004, 0.006, respectively. VBD vs. LGLL, p = 0.041; VBD vs. HTLVt, p = < 0.001; VBD vs. HTLVm, p = < 0.001; VBD vs. HTLVn, p = < 0.002; VBD vs. MS, p = < 0.001; HTLVm vs. HTLVn, p = < 0.001.

**Figure 5:**
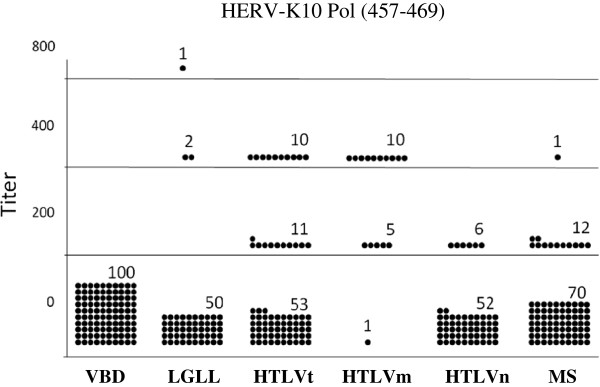
**Distribution of seropositive titers for the anti-HERV-K10 Pol peptide ELISA antibody assay among the various subject populations.** The means for VBD, LGLL, HTLVt, HTLVm, HTLVn and MS subjects were 0, 30, 84, 313, 21, 34, respectively. VBD vs. LGLL, p = 0.041; VBD vs. HTLVt, p = < 0.001; VBD vs. HTLVm, p = < 0.001; VBD vs. HTLVn, p = < 0.002; VBD vs. MS, p = < 0.001; HTLVm vs. HTLVn, p = < 0.001.

Compared to the VBD (0%), LGLL (6%), HTLVt (28%), HTLVm (94%), HTLVn (10%) and MS (16%) patients all had a significantly higher seroprevalence for anti-HERV-K10 Pol antibodies (Table 1). Again, these same relationships held when the results were analyzed for optical density or titer (Figure 4, Figure 5).

When analyzed for seropositivity against both (Gag and Pol together) HERV-K10 peptides, all of the study populations except the LGLL patients (0%) had a significantly higher seroprevalence than the VBD (0%) (Table 2). However, the HTLVm patients had a very high rate (82%), which was significantly higher than the HTLVn subjects (6.9%) and the MS patients (4.8%).

**Table 2:** # of subjects in each population positive for both anti-HERV K10 Gag & Pol antibodies

**Population**	**N**	**# positive (%)**
VBD*	100	0(0)
LGLL*	53	0(0)
HTLVt*	74	18(24)
HTLVm*^+^	16	14(88)
HTLVn*^+^	58	4(7)
MS*^+^	83	4(5)

*p = 1.000, < 0.001, < 0.001, 0.017 and 0.041 for VBD vs LGLL, HTLVt, HTLVm; HTLVn; and MS, respectively.

^+^p = < 0.001 for HTLVm vs HTLVn or vs MS.

Among the HTLVm patients, 8 were HTLV-1 positive and 8 were HTLV-2 positive. Six (75%) of the HTLV-2 positives had antibodies to both HERV-K10 peptides, while all of the 8 (100%) HTLV-1 positives had antibodies to the HERV-K10 Gag peptide, and 7 out of 8 (87.5%) had antibodies to HERV-K10 Pol peptide. Obviously, there were no significant differences in these results. Among the 22 HTLV-1n subjects, 3 (13.6%) were reactive to HERV-K10 Gag peptide, 3 (13.6%) were reactive to HERV-K10 Pol peptide and these 3 (13.6%) were reactive to both peptides. Among the 36 HTLV-2n subjects, 1 (2.8%) was reactive to HERV-K10 Gag, and 3 (8.3%) were reactive to HERV-K10 Pol, with 1 (2.8%) being reactive to both peptides. Again, these differences in HERV-K10 seroprevalences between HTLV-1 and −2 infected subjects were not statistically significant.

The mean OD minus background for the anti-HERV-K10 Gag results among the HTLV-1 m patients was 0.841, while the same value for the HTLV-2 m patients was 0.261 (p = 0.055). The mean titer for the anti-HERV-K10 Gag results among the HTLV-1 m patients was 925, while the same value for the HTLV-2 m patients was 250 (p = 0.129). The mean OD minus background for the anti-HERV-K10 Pol results among the HTLV-1 m patients was 0.236, while the same value for the HTLV-2 m patients was 0.125 (p = 0.072). The mean titer for the anti-HERV-K10 Pol results among the HTLV-1 m patients was 325, while the same value for the HTLV-2 m patients was 300 (p = 0.202).

Table 3 shows the prevalence of HERV-K113 and HERV-K115 genomes among the various populations studied. Seven subjects (6.7%) out of a total of 105 studied were positive for HERV-K113, whereas 18 (17.1%) were positive for HERV-K115 (p = 0.001). The HTLVm patients had a lower frequency of HERV-K113 or HERV-K115 (8%) compared to the VBD (27%), but the difference was not significant (p = 0.160). The HTLVn (27%), LGLL (33%) and MS (33%) subjects had frequencies similar to the VBD.

**Table 3:** Prevalence for HERV-K113 and HERV-K115 in different populations

**Population**	**Total samples**	**HERV-K113+ (%)**	**HERV-K115+ (%)**	**HERV-K113+ (%)**
VBD	30	2(7)	6(20)	8(27)
LGLL	3	1(33)	0(0)	1(33)
HTVLt	24	0(0)	4(17)	4(17)
HTVLm	13	0(0)	1(8)	1(8)
HTVLn	11	0(0)	3(27)	3(27)
MS	24	4(17)	4(17)	8(33)

## Discussion

Increased risk for developing HAM is associated with blood-borne viral transmission, increased proviral DNA loads and anti-HTLV antibodies, polymorphisms in the IL-10 promoter and IL-28B gene, HLA-A*02^-^ and HLA-DRB1*0101 alleles, and impaired anti-HTLV cytotoxic T-cell responses [7,15-19]. There is no indication that either HTLV-1 or HTLV-2 directly infects neuronal tissue. Rather, it is assumed that the central nervous system is collaterally damaged by the anti-viral host immune response [5,8,9]. Presumably, either direct immune system damage or toxic effects of cytokines released in the immune process injure the central nervous system. It would seem plausible that anti-HTLV immunologic cross-reactivity to epitopes within the cells of the central nervous system could explain the tropism and cellular susceptibility to the phenomenon. Indeed, molecular mimicry between HTLV-1 Tax protein and neuron-specific ribonuclear antigen and a cross-reactive immune response has been proposed as part of the pathogenesis of HAM [20].

It has been estimated that 8% of the human genome is comprised of endogenous retroviral elements [21]. Most of these are defective and transcriptionally inactive, but many are capable of viral RNA and protein expression [22-24]. If expressed prior to the ontogeny of the host immune system, they would be recognized as “self”; if subsequent to the formation of a competent immune system, they would be recognized as “foreign” and a potential cause of autoimmune diseases [24,25].

HERV-K10 is a member of the HML-2 class of endogenous retroviruses [26]. It has been shown to be expressed in numerous human tissues including the central nervous system; and it has been a suggested target of autoimmune responses [27,28]. Such a hypothesis is consistent with observations that increased expression of HERV-K in HIV-1 infected patients leads to a cytotoxic T-cell response toward HERV proteins [29-31]. Expression of HERV-K proteins and immune responses thereto have been associated with a variety of human malignancies and autoimmune diseases [32-34]. Further, the HTLV-1 Tax protein has been shown to transactivate HERV-K LTR controlled transcription [35].

Our data indicate that, compared to VBD and HTLV + non-myelopathy subjects, patients with HTLV myelopathy had higher titers of antibodies to two HERV-K10 peptides. This suggests that cross-reactive or direct immunity to HTLV-transactivated HERV-K expression in the central nervous system could play a role in the pathogenesis of HAM. Obviously, however, this data alone would be insufficient to make such a case. If HAM is associated with higher titers of anti-HTLV antibodies, then it is quite possible that these patients coincidentally would have higher antibody titers against HTLV homologous HERV-K10 peptides. However, it is indeed interesting that MS patients, who were HTLV seronegative, also had a statistically significant higher seroprevalence (7.2% vs. 0%) to both HERV-K10 peptides compared to VBD. Recently, results were published that indicate that broad anti-HERV-K Gag and Env-specific T-cell responses were not detected in HTLV-I infected subjects [28]. This would suggest that broad anti-HERV-K immune responses may not be involved in the pathogenesis of HAM; but, it does not negate the possibility that anti-HTLV immune responses, cross-reactive to select HERV-K10 peptides, could. Further testing for a wider range of anti-HERV-K10 immunoreactivity and HERV-K10 expression in HAM and MS patients would seem warranted.

## Methods

### Subjects

In an IRB-approved archival study, serum and peripheral blood mononuclear cells (PBMC) were obtained from 100 VBD, 53 LGLL patients, 74 HTLV-1 or −2 infected individuals (HTLVt), 16 of whom had myelopathy (HTLVm) (8 HTLV-1 and 8 HTLV-2), and 58 who did not (HTLVn) (22 HTLV-1 and 36 HTLV-2), and 83 HTLV negative multiple sclerosis (MS) patients. All LGLL patients had clonal T-cell lymphocytoses, as confirmed by PCR for clonal rearrangements of the T-cell receptor γ- gene and by positivity for CD3, CD8, and CD57 proteins [36]. The HTLV myelopathy patients were all positive for either HTLV-1 or −2 antibodies and DNA in both their peripheral blood and cerebrospinal fluid. They all manifested signs and symptoms of upper motor neuron disease, and met the updated WHO criteria for HTLV myelopathy [37,38]. The MS patients were negative for HTLV-1 and −2 by serologic and nucleic acid studies and fulfilled the McDonald diagnostic criteria, which utilize both clinical and MRI findings [39].

### Serologic studies

All subjects were screened for anti-HTLV antibodies using a commercial HTLV1/2 EIA assay, as previously described [40]. Seropositive individuals were further evaluated using an HTLV-1/2 Western blot, as previously described [41]. Sera were also tested for antibodies against the HERV-K10 Pol peptide REPLENALTVFTD and the HERV-K10 Gag peptide AYENANPECQ (Figure 1). Briefly, the peptides were synthesized (New England Peptide LLC, Gardner MA) and used to immunize rabbits. Pre- and post immune rabbit sera were utilized as positive and negative controls in an enzyme-linked immunoassay (ELISA). The ELISA was performed as follows. The HERV-K10 peptide was diluted in carbonate buffer, pH 9.6 (Sigma, St Louis, MO) and 50 μl of solution containing 0.5 μg of peptide was added to each well and incubated overnight at 4°C. The wells were then washed five times with wash buffer (1x PBS plus 0.2% Tween 20), and 100 μl of superblock blocking buffer (Thermo Scientific, Hudson, NH) was added to each well, incubated for 1 h at 37°C, and washed five times with wash buffer. Then, 50 μl of the primary antibody sample at the desired dilution in diluent buffer was added to the well. A buffer only sample was used as a background control. The samples were incubated for 1 h at 37°C and, then washed five times with wash buffer. Goat anti-rabbit (1:5000) or goat anti-human IgG-HRP (1:2000 in diluent buffer) (Sigma, Saint Louis, MO) was added and incubated for 1 h at 37°C. Plates were then washed five times, and 50 μl of hydrogen peroxide and tetramethylbenzidine was added to each well and incubated for 10 min at room temperature in the dark. The reaction was stopped by the addition of 50 μl 1 N H_2_SO_4_ to each well and the optical densities of the samples were read at 450 nm. A sample was deemed positive if, at a dilution of 1:200, its optical density was ≥ 2.5 x background plus 0.100 for the Gag peptide or ≥ 2.5 x background for the Pol peptide. Multiple permutations of the above conditions were conducted using the pre- and post-immune rabbit sera and a panel of three VBD sera as putative negative controls before settling on the above conditions. Positive samples were then serially diluted and retested to determine their end point titer.

### PCR studies

DNA was extracted from serum or PBMC, and DNA integrity was confirmed by β-globin PCR, as previously described [42]. Only samples that tested positive for human β-globin at 0.1 μg and 1 μg of DNA input were deemed suitable for subsequent retroviral DNA analysis. DNA was analyzed for HTLV-1 and −2 DNA using the *pol* primers SK110/SK111, the HTLV-1 *pol* probe SK-112, and the HTLV-2 *pol* probe SK188, as previously described [42]. Quantification of HTLV viral load was done using real-time PCR, as previously described [13].

The presence or absence of HERV-K113 and HERV-K115 was determined with PCR primers spanning the pre-integration sites and sites within the viral sequences. The primers for HERV-K113 were those used by Moyes et al. and those for HERV-K115 were used by Hughes et al. [12,43]. Conditions for HERV-K113 were as described and those for HERV-K115 were as provided by Hughes et al. [43].

### Statistics

Seroprevalence rates between various subject populations were analyzed using Fisher’s t-test [44]. Comparisons of serologic optical density scores and antibody titers were analyzed using the contingency table method [44].

## Competing interests

The authors declare that they have no competing interests.

## Authors’ contribution

BJP, RP – conceived and designed the experiments. RP, EG, SS, CW, PB, LA, DL – performed the experiments. JG, TL, WS – contributed the samples. BJP, RP – wrote the paper. WS – edited the manuscript. All authors participated in data analysis. All authors read and approved the final manuscript.
